# An injectable self-healing curcumin-sustained-release hydrogel for lower limb ischemia via autophagy inhibition by *Atp6v0d2* downregulation

**DOI:** 10.1093/rb/rbag103

**Published:** 2026-07-02

**Authors:** Hanfei Tang, Fang Wang, Tonglei Han, Xuanyong Liu, Minhui Li, Jiaqi Zhu, Xiao Tang, Guowei Liu, Jiajun Qiu, Daqiao Guo

**Affiliations:** Department of Vascular Surgery, Zhongshan Hospital, Fudan University, Shanghai 200030, China; Institute of Vascular Surgery, Fudan University, Shanghai 200030, China; National Clinical Research Center for Interventional Medicine, Shanghai 200030, China; State Key Laboratory of High Performance Ceramics, Shanghai Institute of Ceramics, Chinese Academy of Sciences, Shanghai 200050, China; Institute of Biomedical Engineering, College of Medicine, Southwest Jiaotong University, Chengdu 610031, China; Department of Vascular Surgery, Zhongshan Hospital, Fudan University, Shanghai 200030, China; Institute of Vascular Surgery, Fudan University, Shanghai 200030, China; National Clinical Research Center for Interventional Medicine, Shanghai 200030, China; State Key Laboratory of High Performance Ceramics, Shanghai Institute of Ceramics, Chinese Academy of Sciences, Shanghai 200050, China; Department of Vascular Surgery, Zhongshan Hospital, Fudan University, Shanghai 200030, China; Institute of Vascular Surgery, Fudan University, Shanghai 200030, China; National Clinical Research Center for Interventional Medicine, Shanghai 200030, China; Department of Vascular Surgery, Zhongshan Hospital, Fudan University, Shanghai 200030, China; National Clinical Research Center for Interventional Medicine, Shanghai 200030, China; Department of Vascular Surgery, Zhongshan Hospital, Fudan University, Shanghai 200030, China; Institute of Vascular Surgery, Fudan University, Shanghai 200030, China; National Clinical Research Center for Interventional Medicine, Shanghai 200030, China; Department of General Surgery, Shanghai Xuhui Central Hospital, Shanghai 200030, China; State Key Laboratory of High Performance Ceramics, Shanghai Institute of Ceramics, Chinese Academy of Sciences, Shanghai 200050, China; Department of Vascular Surgery, Zhongshan Hospital, Fudan University, Shanghai 200030, China; Institute of Vascular Surgery, Fudan University, Shanghai 200030, China; National Clinical Research Center for Interventional Medicine, Shanghai 200030, China

**Keywords:** peripheral artery disease, hydrogel, muscle necrosis, fibrosis, curcumin

## Abstract

Although hydrophobic drugs demonstrate the promising therapeutic efficacy for peripheral artery disease *in vitro*, their *in vivo* performance is often hindered by low bioavailability due to inherent hydrophobicity. To address this, an injectable, self-healing and curcumin-sustained-release hydrogel (CGP hydrogel) using a gelatin/polyvinyl alcohol hydrogel (GP hydrogel) was developed. The CGP hydrogel effectively preserved the DPPH radical-scavenging activity of curcumin at room temperature. Meanwhile, in the murine hindlimb ischemia model, a single intramuscular injection of CGP following femoral artery ligation significantly improved the motor function of the ischemic limb compared to curcumin suspension. This superior therapeutic outcome is attributed to the sustained release of curcumin from the CGP hydrogel. Mechanistically, we found that CGP, but not GP, inhibited the expression of *Atp6v0d2* in ischemic skeletal muscle cells and suppressed ischemia-induced autophagy. The inhibition of autophagy may mitigate tissue and cellular necrosis and promote the expression of Myogenin. Furthermore, CGP reduced the expression of pro-fibrotic factors such as *Tgfb1* and *Ctgf* and prevented skeletal muscle fibrosis. In summary, the CGP hydrogel, through its sustained release of curcumin, inhibits ischemia-induced autophagy, alleviates necrosis and fibrosis in skeletal muscle tissue and preserves motor function in the ischemic limb.

## Graphical Abstract

**Graphical Abstract:**
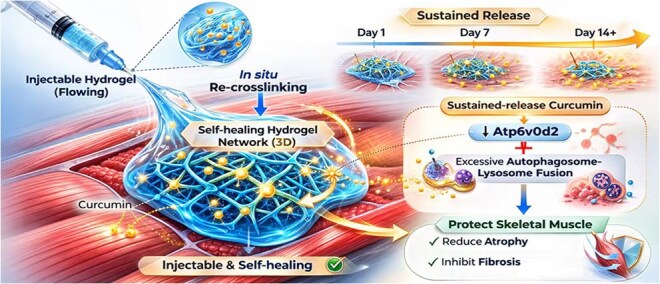

## Introduction

Peripheral artery disease (PAD) is one of the atherosclerotic diseases, which exhibits a rising global prevalence [1]. PAD often presents with an insidious onset and prolonged progression, frequently resulting in delayed diagnosis and treatment [2]. Approximately 11% of patients with PAD develop rest pain, affecting about 1.3% of individuals aged 40 years or older [3]. Severe progression of PAD may result in amputation or even mortality. Current treatment strategies include pharmacological therapy and surgical interventions. Surgical interventions include artificial vascular bypass and endovascular stenting. The former is highly invasive, while the latter is associated with poor long-term patency rates [4, 5]. Pharmacotherapy, integral throughout treatment, involves anticoagulants, antiplatelet agents and lipid-lowering drugs [6]. These medications help mitigate symptom progression or prevent postoperative re-occlusion by inhibiting thrombus formation or slowing the advancement of atherosclerosis.

Although current revascularization therapies represent the etiological treatment for PAD, limited attention has been paid to accelerating the recovery of motor function in the ischemic skeletal muscle. Supervised high-intensity home-based walking has been shown to improve motor function and blood flow perfusion in the ischemic limb [7, 8]. However, such therapy frequently induces intermittent claudication, leading to poor patient compliance. Moreover, it is also unsuitable for PAD that has progressed to chronic limb-threatening ischemia.

As another ischemic cardiovascular disease, studies in myocardial infarction models have found that the application of curcumin can improve left ventricular ejection fraction and mitigate myocardial fibrosis [9]. Although curcumin exhibits notable protective effects on ischemic myocardium, its hydrophobicity and short pharmacokinetic profile have largely confined its clinical application to digestive system disorders such as inflammatory bowel disease [10, 11].

To enhance the aqueous solubility of curcumin, L-arginine has been employed to form a complex with weakly acidic curcumin, increasing its solubility 440-fold [12]. However, this approach does not contribute to improving its pharmacokinetic profile. To achieve sustained release, curcumin can be encapsulated in soybean protein to form nanoparticles [10]; nevertheless, the *in vivo* application of plant proteins poses unpredictable risks of allergic reactions.

Therefore, to improve the bioavailability and pharmacokinetic profile of curcumin, this study employs gelatin/polyvinyl alcohol (PVA) hydrogels for curcumin loading. We demonstrate that this injectable self-healing curcumin-sustained-release hydrogel effectively preserves muscle function during limb ischemia and inhibits skeletal muscle fibrosis. Furthermore, integrated analysis of RNA-seq data from murine hindlimb ischemia models, together with subsequent experimental validation, revealed that curcumin can downregulate Atp6v0d2 expression, suppress ischemia-induced autophagy and inhibit necrosis and fibrosis in ischemic skeletal muscle, ultimately preserving muscle function.

## Materials and methods

### Synthesis of GP CGP composite hydrogels

Fifteen percent (w/v) gelatin (Gelatin from cold water fish skin, Sigma-Aldrich, USA) was completely dissolved in ultrapure water with an ice bath for 4 h [13]. Poly(vinyl alcohol; PVA1799; Ourchem, China) was dissolved in ultrapure water at a concentration of 15% (w/v) and stirred at 98°C for 1 h. The gelatin/PVA (GP) sol was subsequently formed by mixing 7 mL of the gelatin solution with 3 mL of the PVA solution under magnetic stirring for 1 h.

Crosslinking was initiated by adding 1.5% glutaraldehyde (GA) (SCR, Shanghai) and 0.01 M sodium borate decahydrate (SCR, Shanghai) into the solution at a sol-to-crosslinker volume ratio of 10:1, followed by rapid stirring. The mixture was then placed into an oven at 65°C for 20 min to form GP hydrogels. For curcumin-loaded GP (CGP) hydrogels, 50 mg of curcumin was directly dispersed into 100 mL of the GP hydrogel and mixed thoroughly. To neutralize residual GA, a 10-min incubation was performed by immersing the hydrogels in 50 mL of 0.1 M glycine (Aladdin, Shanghai), after which they were rinsed three times with ultrapure water.

The curcumin loading capacity was assessed by immersing CGP hydrogels in ethanol and then centrifuging (12 000 rpm, 10 min) to achieve complete release of the encapsulated curcumin. The concentration of released curcumin was determined by measuring its absorbance at 430 nm on a multifunctional microplate reader, followed by quantification using a standard curve established at the same wavelength (*y* = 30.18*x* − 1.85, *R*^2^ = 0.999). Curcumin loading reached approximately 350 µg per 1 mL of CGP hydrogel, giving a loading capacity of 350 µg/mL. Finally, both GP and CGP hydrogels were subjected to a single freeze-thaw cycle at −80°C to obtain the final products.

### Sample characterization

Scanning electron microscopy (S-3400N, HITACHI, Japan) was employed to image hydrogel morphology. A diffractometer (D2PHASE, BRUKER, USA) was used to acquire X-ray diffraction (XRD) patterns. Absorption peaks of the hydrogel were measured by a Fourier Transform Infrared (FTIR) spectrometer (Tensor 27, Bruker, Germany) over the wave range at 400–4000 cm^−1^. A Ultraviolet-Visible (UV-Vis) spectrophotometer (Lambda 750, PerkinElmer, UK) was employed to acquire the UV–Vis absorption spectra. Photoluminescence (PL) spectra were measured over a wavelength range of 400–700 nm using a multifunctional microplate reader (BioTek Cytation 5, USA) under an excitation wavelength of 380 nm.

### Rheological measurements

Rheological measurements of the CGP composite hydrogels were carried out on a rheometer (MCR 301, Anton Parr, Austria) at 25°C, using samples with a diameter of 20 mm. The frequency-dependent storage modulus (*G*′) and loss modulus (*G*″) were tested from 0.1 to 100 Hz. Strain-dependent modulus variations were then assessed from 1% to 1000% to evaluate the mechanical structural stability of the hydrogels. Additionally, cyclic strain testing from 1% to 500% for three cycles was performed to evaluate the self-healing performance of CGP hydrogels.

### Self-healing and injectability performance testing

After being cut and left to reassemble for 5 min, the GP and CGP hydrogels were grasped by tweezers to evaluate their morphological stability. The GP and CGP hydrogels were injected through a syringe into a Petri dish to form the letters ‘GEL’.

### Curcumin release

One millilitre of CGP hydrogel was incubated at 37°C in 5 mL of phosphate-buffered saline (PBS, pH = 7.4). Release medium was sampled at designated time points, and fresh PBS of equal volume was added to maintain sink conditions. Absorbance of the released curcumin was measured at 430 nm using a multifunctional microplate reader (BioTek Cytation 5, USA). Concentrations were then determined from a standard curve established at the same wavelength, allowing calculation of the cumulative release.

### DPPH scavenging behavior

1,1-diphenyl-2-picrylhydrazyl (DPPH) was dissolved in ethanol to obtain a 1 mM DPPH solution. Then, 100 µL of the DPPH solution was mixed with 100 µL of GP hydrogel (10 μL, 20 μL, 50 μL or 100 μL of CGP hydrogels) and 800 µL of ethanol. After thorough mixing, the system reacted at 37°C. In the control group, the hydrogel was replaced with an equal volume of ultrapure water. Following centrifugation at 1000 rpm for 5 min, the supernatant was collected and added into a 96-well microplate. The absorbance at 517 nm was recorded using a multifunctional microplate reader. The following equation was used to calculate the DPPH scavenging rate:


$$\mathrm{DPPH}\;\mathrm{scavenging}\;(\%)=(1-\frac{A_{S}}{A_{C}})\times 100\%,$$

where *A_s_* is the absorbance of the experimental group and *A_c_* that of the control group, both measured at 517 nm.

### Cell culture and preparation of hydrogel extract

Endothelial cell medium (ECM, ScienCell, USA) supplemented with 5% (v/v) fetal bovine serum (FBS) and 1% (v/v) endothelial cell growth supplement/heparin was used to culture human umbilical vein endothelial cells (HUVECs). DMEM/F12 medium (Keygentec, China) with 5% (v/v) FBS was used to culture mouse myoblasts (C2C12). Only cells from passages 7 to 10 were used in this study.

Hydrogel extracts were prepared by soaking CGP or GP hydrogels in serum-free medium specific to each cell type, with a 1:200 (v/v) ratio. After 12 h, the suspension was then centrifuged (3000 rpm, 1 min) and the resulting supernatant was used as the hydrogel extract.

### Cell viability assay

In 96-well plates, cells were seeded at approximately 5 × 10^4^ cells/well. Following adherence, the cells were exposed to hydrogel extracts that had been prepared in the respective complete medium for each cell type. Cells were maintained for 5 days, with the culture medium replaced every other day. On Days 1, 3 and 5, cell viability was evaluated using a CCK-8 assay kit, following the manufacturer’s instructions.

### Wound healing assay

After being seeded into 6-well plates at 1.2 × 10^6^ cells/well, cells were cultured to roughly 90% confluency. Thereafter, cells were cultured in serum-deprived medium for 24 h to synchronize their cells cycle. A 200 µL pipette tip was used to create uniform vertical scratches across the cell monolayer, followed by gentle washing of the wells with PBS to clear away detached cells. Hydrogel extracts prepared in the corresponding serum-free medium for each cell type were then added into the corresponding group. The following equation was used to calculate migration rates:


$$\text{Migration}\;\text{rate}\;(\%)=(1-\frac{\;\mathrm{final}\;\mathrm{width}}{\mathrm{initial}\;\mathrm{width}})\times 100\%,$$

where initial width and final width are the width of the scratch quantified using ImageJ from bright-field images at designated time points.

### Capillary tube formation assay

In Matrigel-coated (Corning, USA) 96-well plates, HUVECs were seeded at approximately 2–3 × 10^4^ cells/well. Following exposure to hydrogel extracts prepared in ECM, capillary-like structure formation was observed under an optical microscope (Leica) at designated time points. The number of master junctions, serving as an indicator of *in vitro* angiogenic potential, was quantified by the Angiogenesis Analyzer in ImageJ.

### Cell culture in conditions with oxygen-glucose deprivation

In 6-well plates, C2C12 were plated at approximately 1.2 × 10^6^ cells/well. Following adherence, cells were assigned to five groups: NC (normal DMEM/F12), PC (glucose-free DMEM/F12), GP (GP extract from glucose-free DMEM/F12), Baf. (glucose-free DMEM/F12 with 100 nM Bafilomycin A1) and CGP (CGP extract from glucose-free DMEM/F12). Except for the NC group, all other groups were incubated for 8 h under 1% O_2_ and 5% CO_2_ at 37°C to induce hypoxic injury.

### Calcein/PI staining

After C2C12 cells were cultured under oxygen-glucose deprivation (OGD) conditions for 8 h, both the supernatant and adherent cells were collected and resuspended in PBS and combined into a single tube. Live cells and dead cells were labeled with Calcein-AM and propidium iodide, respectively. (Ptoteintech, China) Flow cytometry was used to analyzed the live/dead cell ratio.

### Transfection of pCMV-mCherry-GFP-Lc3 plasmid

For transfection, cells were transfected with pCMV-mCherry-GFP-Lc3 (Beyotime, D2816) by lipofectamine 3000 (Thermofisher, America). After transfection, these cells were cultured in conditions under OGD conditions for 8 h. Finally, laser confocal scanning microscopy (Nikon, 100×) was used to image the transfected cells.

### Establishment of murine hindlimb ischemia model

Animal experiments were approved by the Animal Ethics Committee of Zhongshan Hospital Affiliated to Fudan University (approval numbers: 2021-094) and followed the NIH Guide for the Care and Use of Laboratory Animals (8th Edition, 2011). Male C57BL/6 mice (6 weeks old; Shanghai Laboratory Animal Co., Ltd.) were anesthetized via intraperitoneal administration of 1% pentobarbital at 50 μL per 10 g body weight. After shaving and disinfecting the target hindlimb, the femoral artery was exposed through a longitudinal incision in the inguinal region, then ligated at proximal, distal and branch points using 8-0 silk sutures and subsequently transected. The animals were divided into 6 groups: sham-operated (NC), positive control (PC), curcumin suspension given intraperitoneally (CSP) or intramuscularly (CSM), GP and CGP. On postoperative Day 1, each mouse received a 150 μL injection (saline, curcumin suspension, GP or CGP) distributed across 4 hindlimb sites (adductor, quadriceps and gastrocnemius medial/lateral heads). On Day 28, the gastrocnemius muscle from the treated side was harvested for subsequent assays.

### Open field testing in mice with hindlimb ischemia

Bilateral femoral artery ligation was chosen for C57BL/6 mice due to their strong collateral circulation capacity. Open field testing was conducted on postoperative Day 28. In this test, each animal was removed from its home cage and positioned in the central zone facing away from the experimenter, after which the experimenter exited and the chamber door was closed to permit free exploration of mice [14]. Video recordings captured locomotor activity over the 0–15 min interval.

### Blood flow perfusion in murine hindlimb ischemia model

On postoperative Days 1, 4, 7, 14 and 28, mice in the NC, PC, GP and CGP groups, all of which underwent left hindlimb ischemia, were anesthetized and depilated before being positioned on a heating pad maintained at 37°C. To measure absolute blood flow in both hindlimbs, laser Doppler perfusion imaging (LDPI, Moor Instruments, Devon, Sweden) was employed, from which the relative blood flow ratio (ligated side/nonligated side) in the ischemic ankle and foot was calculated [14].

### Histological analysis

Tissue collection was performed on postoperative Days 4, 7 and 28. Following euthanasia by anesthetic overdose, the gastrocnemius muscles were excised and immediately placed in 4% paraformaldehyde for 24 h. The specimens were subsequently sectioned at 5 μm after paraffin embedding. Hematoxylin and eosin staining (H&E, Servicebio, China) and Masson’s trichrome staining (Servicebio, China) were used for histological evaluation and fibrosis assessment. Apoptotic cells were detected with the DAB (SA-HRP) TUNEL Cell Apoptosis Detection Kit (Servicebio, China), and quantitative evaluation of stained sections was conducted using ImageJ.

### Immunohistochemistry

Tissue sections for immunohistochemistry (IHC) and immunofluorescence (IF) were deparaffinized, rehydrated, and then treated with EDTA for antigen retrieval. For IHC, endogenous peroxidases were blocked with peroxidase blocking solution, followed by blocking with 1% bovine serum albumin (BSA) at room temperature for 30 min. The sections were then incubated with anti-Atp6v0d2 antibody (1:200, Abcam) at 4°C overnight. After PBS washing, HRP-conjugated goat anti-rabbit secondary antibody was applied at room temperature for 30 min, and signals were developed using DAB substrate before counterstaining with hematoxylin. For IF, 0.1% Triton X-100 was applied to the sections for permeabilization after antigen retrieval. Sections were then washed with PBS and incubated with primary antibodies against P62 (1:200) & Fibronectin (1:200), CD31 (1:100) & α-SMA (1:500) (all from Proteintech) in 1% BSA at 4°C overnight. On the next day, after washing, sections were incubated with fluorescent secondary antibodies at room temperature for 90 min, followed by mounting using Antifade Mounting Medium containing DAPI (Beyotime Inc.). Confocal microscopy was used for image acquisition.

### Western blot analysis

On postoperative Days 4, 7 and 28, the gastrocnemius muscles from the ischemic hindlimbs were harvested and stored at −80°C. RIPA buffer (Beyotime, China) and protease inhibitors (Yeason, China) were used for protein extraction from both OGD-treated C2C12 cells and these muscle tissues according to the protocol of the manufacturer. After centrifugation (12 000 × *g*, 10 min) at 4°C, this supernatant was collected and mixed with loading buffer followed by denaturing for 10 min at 99°C prior to SDS-PAGE. After electrophoresis, PVDF membranes, to which proteins were transferred, were blocked with 10% nonfat dried milk and washed with TBST. After overnight incubation with primary antibodies at 4°C, membranes were incubated with HRP-conjugated secondary antibodies for 2 h at room temperature, and protein bands were then detected by ECL chemiluminescence. The following primary antibodies were employed: Myod (1:2000, Proteintech), Myog (myogenin, 1:5000, Proteintech), Atp6v0d2 (1:1000), Lc3 (1:4000, Proteintech), P62 (1:5000), Tgfb1 (1:1000, Proteintech), Ctgf (1:2000, Proteintech) and Fibronectin (1:4000).

### RT-qPCR

RNA isolation from C2C12 cells was performed using the Cell/Tissue Total RNA Kit (Yeason, China), followed by reverse transcription into cDNA using the AdvanceFast 1st Strand cDNA Synthesis Kit (Yeason, China). The QuantStudio Real-Time PCR System (Thermo Fisher) was used to perform RT‐qPCR according to the manufacturer’s amplification protocol. β-Actin was used as the housekeeping gene, and expression levels were determined via the 2^–ΔΔCt^ method. Detailed primer information can be found in Supplementary Table S1.

### RNA-seq

RNA samples were prepared according to the manufacturer’s instructions (Illumina, San Diego, CA, USA). Libraries were constructed from total RNA with the TruSeq Stranded mRNA Library Prep Kit (Illumina). Nanodrop was used to determine RNA concentration and integrity. The size of all libraries was about 300 bp. Quality control of the libraries was confirmed by RNA size analysis on an Agilent 4200 Tapestation RNA Screentape. Sequencing was performed in paired-end mode with 150 cycles on the 500/550 High Output NextSeq platform (Illumina). Variant calling was conducted using the Genome Analysis Toolkit (GATK, v4.2.4.1) Best Practices workflow, followed by annotation of variant call format files with Variant Effect Predictor (VEP, v.104).

### Statistical analysis

All statistical analyses, including analyses of the results of RNA-seq, were performed using R software. All quantitative results with independent biological replicates are expressed as mean ± standard error (Mean ± SE). Statistical analysis involved unpaired two-tailed Student’s *t*-tests for two-group comparisons and two-way analysis of variance (ANOVA) for multi-group comparisons. Statistical significance was set at *P* < 0.05. (* *P* < 0.05, ** *P* < 0.01, *** *P* < 0.001).

## Results and discussion

### Construction and characterization of GP and CGP hydrogels

**Figure 1:**
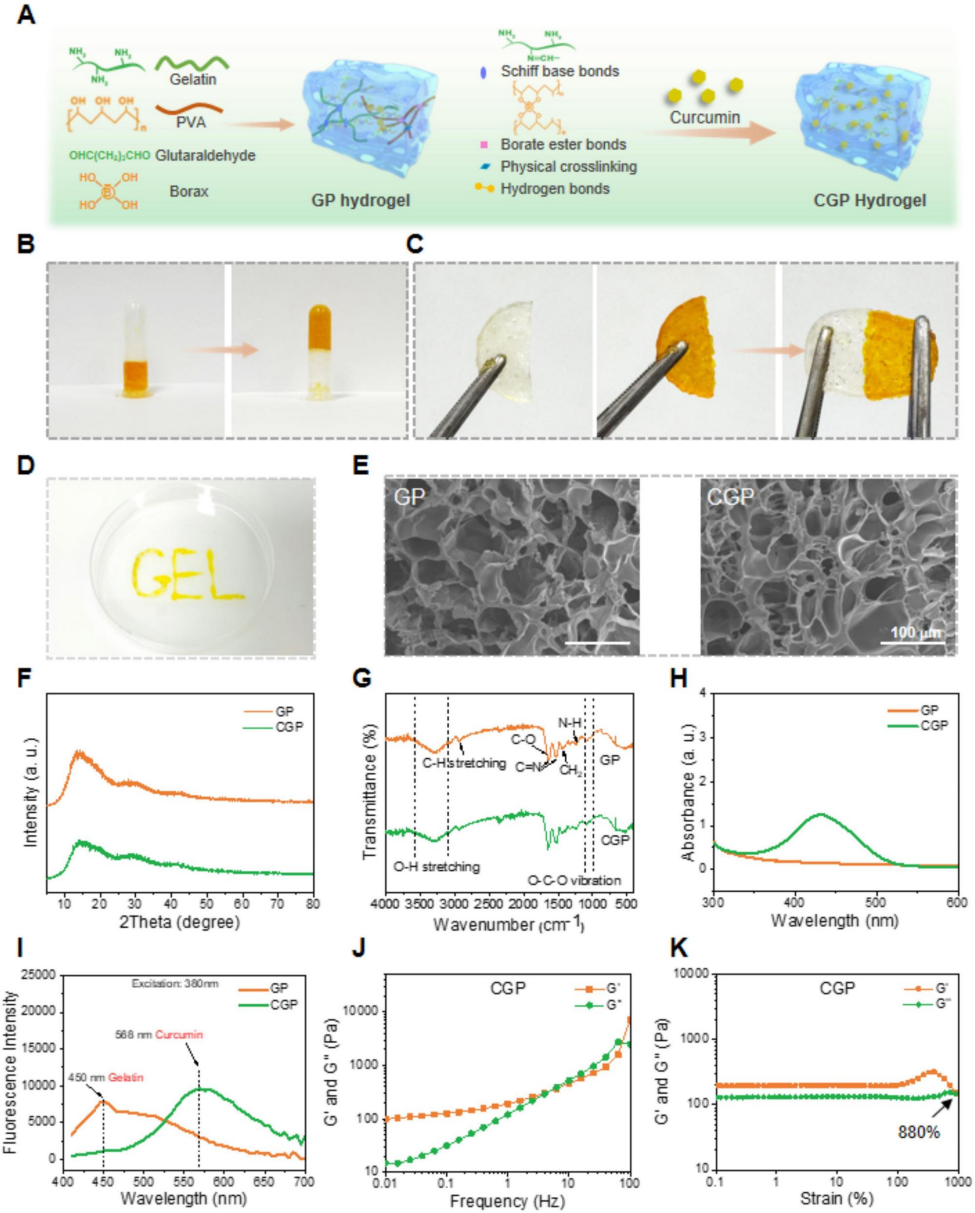
Fabrication and characterization of GP and CGP hydrogels. (**A**) The synthesis process of the GP hydrogel and CGP hydrogel. Representative photographs of the (**B**) sol-gel transformation, (**C**) self-healing and (**D**) injectability of the CGP hydrogel. (**E**) Representative SEM images of internal structures, (**F**) XRD patterns, (**G**) FTIR spectra, (**H**) UV–Vis absorption spectra and (**I**) fluorescence spectra of GP and CGP hydrogels. The *G*′ and *G*″ of the CGP hydrogel (**J**) in the frequency range of 0.1–100 Hz and (**K**) at strains ranging from 1% to 1000%.

As depicted in Figure 1A, gelatin and PVA were employed to fabricate dual-network gelatin/PVA-based (GP) hydrogel and curcumin-loaded gelatin/PVA-based (CGP) hydrogel via physicochemical crosslinking. Gelatin, a polypeptide derived from collagen, contains RGD motifs, which play a pivotal role in promoting cell adhesion [15]. PVA is a synthetic polymer known for its biocompatibility and elasticity [16]. The chemical crosslinking network was formed through two primary mechanisms: Schiff base bonds between the aldehyde groups of GA and the free amino groups (-NH_2_) of gelatin, as well as diol-borate complexes between borax and PVA [13]. Additionally, aldol condensation occurred between the hydroxyl groups of PVA and the aldehyde groups of GA. Physical crosslinking, on the other hand, occurred during the freeze-thaw processing of the PVA molecules. The formation of numerous hydrogen bonds together with molecular entanglement contributed to this effect, substantially reinforcing the hydrogel networks. Finally, the hydrophobic curcumin was incorporated into the hydrogels, facilitated by the amphiphilicity of both gelatin and PVA [17, 18].

The sol-gel transition was clearly observed in both the GP and CGP hydrogels, as shown in Figure 1B and Supplementary Figure S1. The GP and CGP hydrogels possess self-healing and injectable properties, owing to the diol-borate ester bonds, reversible Schiff base linkages and hydrogen bonds within their crosslinking network (Figure 1C and D and Supplementary Figures S2 and S3). Hydrogels featuring reversible crosslinking exhibit dynamic network architectures. These architectures enable the hydrogels to break apart under the shear forces encountered during injection. Once the forces are removed, the hydrogels can then reassemble themselves [19]. Consequently, self-healing hydrogels can be injected as numerous solid fragments, which then coalesce into an integral gel at the target site [20]. Furthermore, self-healing can improve the constant release of drugs and prolong the lifespan of the hydrogel network under mechanical force, especially in the motion system including muscle, tendon and bone [20, 21].

A clear yellow hue was observed in the CGP hydrogel due to the homogeneous dispersion of curcumin throughout the hydrogels. This visual characteristic serves as evidence that curcumin molecules have been successfully incorporated into the CGP hydrogels, which demonstrate excellent dispersibility. Figure 1E illustrated the internal structures of the GP and CGP hydrogels. The GP hydrogel featured smooth-walled pores, with an average diameter of around 100 µm. When it comes to the CGP hydrogel, which was loaded with curcumin, its internal structure showed no substantial differences compared to that of the GP hydrogel. This indicated that curcumin is evenly distributed within the hydrogel matrix, thereby contributing to the formation of a stable structure.

Broad peaks at 2θ  =  14.3°, 28.2° and 40.6° were observed in the XRD patterns of GP and CGP hydrogel (Figure 1F), which corresponded to the characteristic peaks of gelatin and PVA [22, 23]. As shown in Figure 1G, a wide absorption band in FTIR spectra over the 3080–3580 cm-^1^ range, related to O-H stretching vibrations, was observed for both GP and CGP hydrogels. This band stems from the intramolecular and intermolecular hydrogen bonds between gelatin and PVA. C-H stretching vibrations of the aliphatic hydrocarbon chain account for the absorption peak at 2935 cm^−1^, while CH_2_ bending vibrations are associated with the peak at 1430 cm^−1^. The formation of imine (C=N) bonds is indicated by characteristic peaks at 1630–1650 cm^−1^ and 1535–1550 cm^−1^, corresponding to Schiff base linkages formed between the amino groups of gelatin and aldehyde groups in GA crosslinking agents.

It is worth noting that the broad peak around 1630 cm^−1^ may coincide with a C-O stretching vibration peak near 1670 cm^−1^, due to the borate ester linkages related to PVA crosslinking [24]. Moreover, the characteristic peak at 1230 cm^−1^ corresponds to the N-H bending vibration [25], and the peak in the 990–1100 cm^−1^ range corresponds to the O-C-O vibration band. It reveals that the formation of the GP and CGP hydrogels mainly encompasses Schiff base linkages between gelatin and GA, borate ester bonds between PVA and borax, and a substantial quantity of intermolecular and intramolecular hydrogen bonding interactions among gelatin and PVA molecules. Curcumin exhibits characteristic peaks of O-H (3511 cm^−1^), C-O and C-C ring (1450–1630 cm^−1^) and C-O-C functional groups (1000–1300 cm^−1^) [26]. However, owing to the overlap of these peaks with those associated with the hydrogel matrix and the low proportion of curcumin within the CGP hydrogel, the infrared characteristic peaks corresponding to curcumin are barely discernible. The GP hydrogel had no obvious ultraviolet absorption peak in the UV–Vis absorption spectra (Figure 1H). The CGP hydrogel, by contrast, showed a distinct absorption peak at 430 nm that matched the π-π* transition of curcumin, indicating that curcumin has been successfully loaded into the CGP hydrogel (Figure 1H). Furthermore, under excitation at 380 nm, the photoluminescence (PL) spectra of the GP and CGP hydrogels were evaluated, and the results are presented in Figure 1I. Both the GP and CGP hydrogels exhibited a PL peak at 450 nm, which matched the fluorescence characteristics of tyrosine and tryptophan in gelatin [27]. Significantly, the CGP hydrogel showed a distinct peak at 568 nm, which was consistent with the characteristic PL peak of curcumin [28].

The *G*′ and *G*″ of CGP hydrogel exhibited frequency-dependent behavior over 0.1–100 Hz (Figure 1J). Within the 0.1–1 Hz, *G*′ was significantly higher than *G*″. As the frequency increased, *G*′ and *G*″ gradually converge, intersecting at approximately 10 Hz. As shown in Figure 1K, the moduli of CGP hydrogels varied within strain from 1% to 1000%. The gel state was maintained at strains below 880%, where *G*′ > *G*″. Upon exceeding this critical strain of 880%, *G*′ < *G*″, and the hydrogel underwent a gel-to-sol transition, marked by a steep decline in both *G*′ and *G*″. The rheological properties of GP hydrogel were similar to those of the CGP hydrogel (Supplementary Figure S4). These results demonstrate the excellent gel-state maintenance capability of the CGP hydrogel, which can be attributed to the synergistic effect of chemical and physical crosslinking in the gelatin/PVA network [29].

### Curcumin-release and DPPH scavenging behavior of the CGP hydrogel

**Figure 2:**
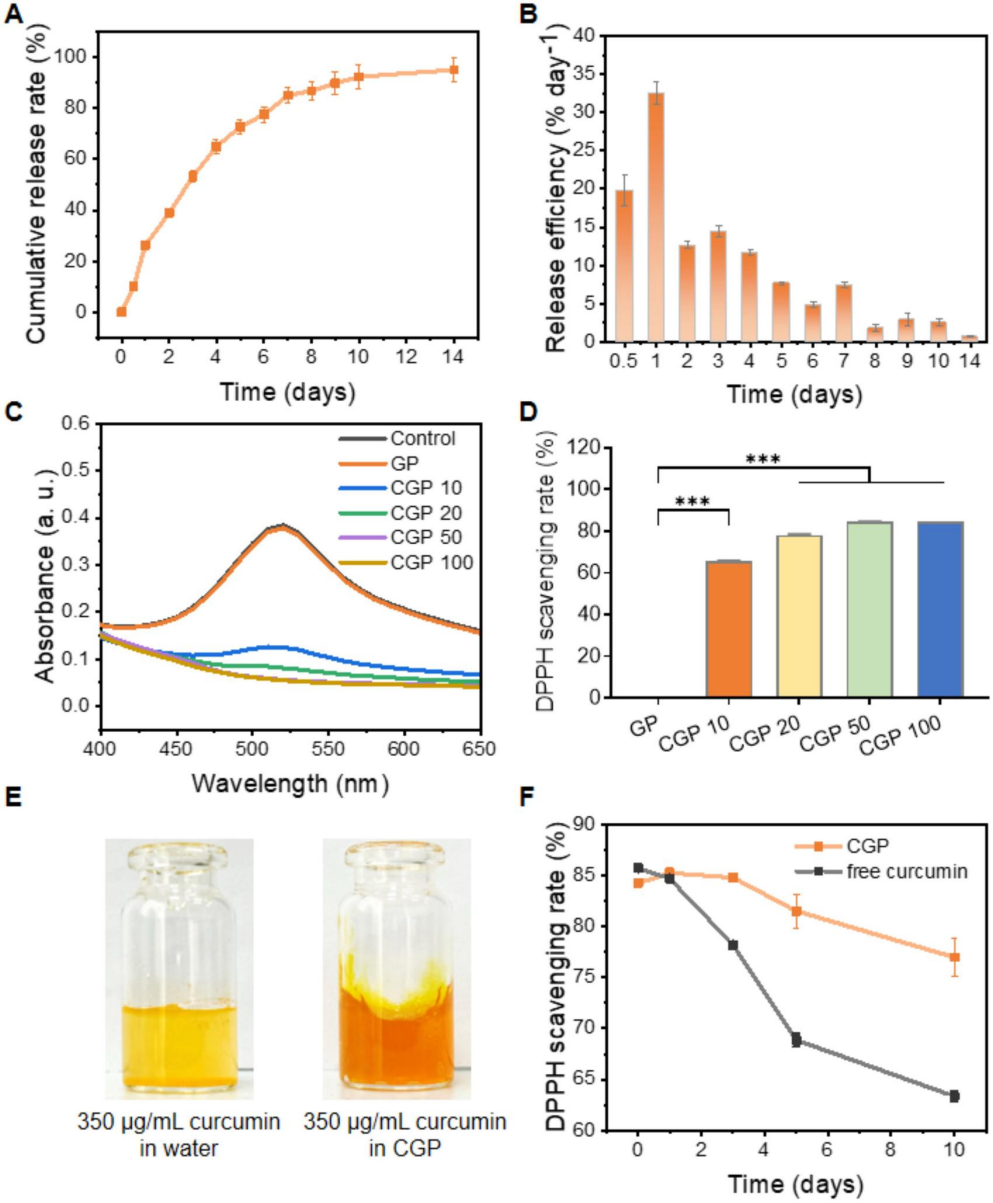
Curcumin-release and DPPH scavenging behavior of the CGP hydrogel. (**A**) Curcumin release curve and (**B**) release efficiency of the CGP hydrogel within 14 days. (**C**) UV–Vis spectra of DPPH and DPPH treated with 100 μL of GP hydrogel, as well as 10 μL, 20 μL, 50 μL and 100 μL of CGP hydrogels. (**D**) Bar graph of DPPH scavenging rate after treatment with 100 μL of GP hydrogel and 10 μL, 20 μL, 50 μL and 100 μL of CGP hydrogels employed *t*-test. (**E**) Photographs of curcumin in water and in the GP hydrogel at a concentration of 350 μg/mL. (**F**) DPPH scavenging capacity comparison between the CGP hydrogel and a free curcumin solution containing the same dose of curcumin within 10 days. (Data was expressed as mean ± SE; * *P* < 0.05, ** *P* < 0.01, *** *P* < 0.001; *n* = 6.)

The release behavior of curcumin from CGP hydrogels was assessed in PBS at 37°C. The cumulative release profile was determined based on the standard curve in Supplementary Figure S5, and the curcumin release behavior at different time points is illustrated in Figure 2A. The CGP hydrogel exhibited a gradual and continuous release of curcumin, with approximately 95% of the loaded curcumin released within 14 days, indicating a sustained-release profile. The release rate increased rapidly from 12 h to 24 h, reaching a maximum on the first day, and then gradually decreased (Figure 2B).

Excessive oxidative stress is one of the key pathological factors under the OGD conditions. Designing hydrogels capable of mitigating this stress has emerged as an effective approach to protect cells from the detrimental effects inflicted by such stress. Herein, DPPH was a model free radical to evaluate antioxidant activity. DPPH exhibits strong absorption at 517 nm, and the absorbance decreases proportionally after transferring an electron or a hydrogen atom to the odd electron in DPPH [30]. When incubated with GP hydrogel (100 μL), CGP 10 (10 μL), CGP 20 (20 μL), CGP 50 (50 μL) and CGP 100 (100 μL) hydrogels, the characteristic absorption peak of DPPH gradually diminished (Figure 2C). As shown in Supplementary Figure S6, curcumin suspensions and CGP hydrogels containing an equivalent dose of curcumin exhibited similar DPPH scavenging effects, indicating that the biochemical properties of curcumin loaded in CGP hydrogel were not substantially altered. For the DPPH assay, both hydrogel and curcumin suspension were placed in a 90% ethanol solution, which significantly increased the solubility of curcumin [31], thereby allowing it to react completely with DPPH. The GP hydrogel showed negligible DPPH scavenging activity, whereas 10 μL, 20 μL, 50 μL and 100 μL of CGP hydrogels scavenged 65.6%, 78.2%, 84.6% and 84.8% of DPPH, respectively (Figure 2D). The antioxidant capacity of CGP hydrogel gradually increased with the dosage of CGP and showed a marginal effect at 50 μL.

As depicted in Figure 2E, curcumin at a concentration of 350 µg mL^−1^ cannot be completely dissolved in water. The solubility of curcumin in water is only 2.792 μg mL^−1^ [32]. The poor water solubility of curcumin significantly hampers its bioavailability, thereby presenting a notable obstacle to its effective utilization. In contrast, curcumin at the same concentration can be uniformly dispersed within the GP hydrogel, resulting in the formation of curcumin-loaded CGP hydrogels (Figure 2E). For antioxidant stability evaluation, the CGP hydrogel and a free curcumin suspension with equal dosage of curcumin were kept at 37°C for 10 days. Antioxidant activities were monitored at designated time intervals (Figure 2F). At the end of the 10-day period, the DPPH scavenging capacity of the CGP hydrogel remained almost at its initial level. In stark contrast, the activity of free curcumin exhibited a significant decrease, with the DPPH scavenging rate declining to 62.3% by Day 10. The protective effects of hydrogel on the antioxidant ability of curcumin may result from the biomimetic structure, soft porous microarchitecture and favorable biomechanical properties [33].

### Effect of CGP on cell viability of C2C12, HASMCs and HUVECs

The application of CGP must prioritize safety considerations alongside its efficacy in protecting ischemic limbs. Current studies indicate that curcumin inhibits the proliferation, migration and survival of cancer cells and suppresses tube formation of HUVECs in tumor-conditioned media [34–37]. However, research on its safety profile in normal cells remains limited.

**Figure 3:**
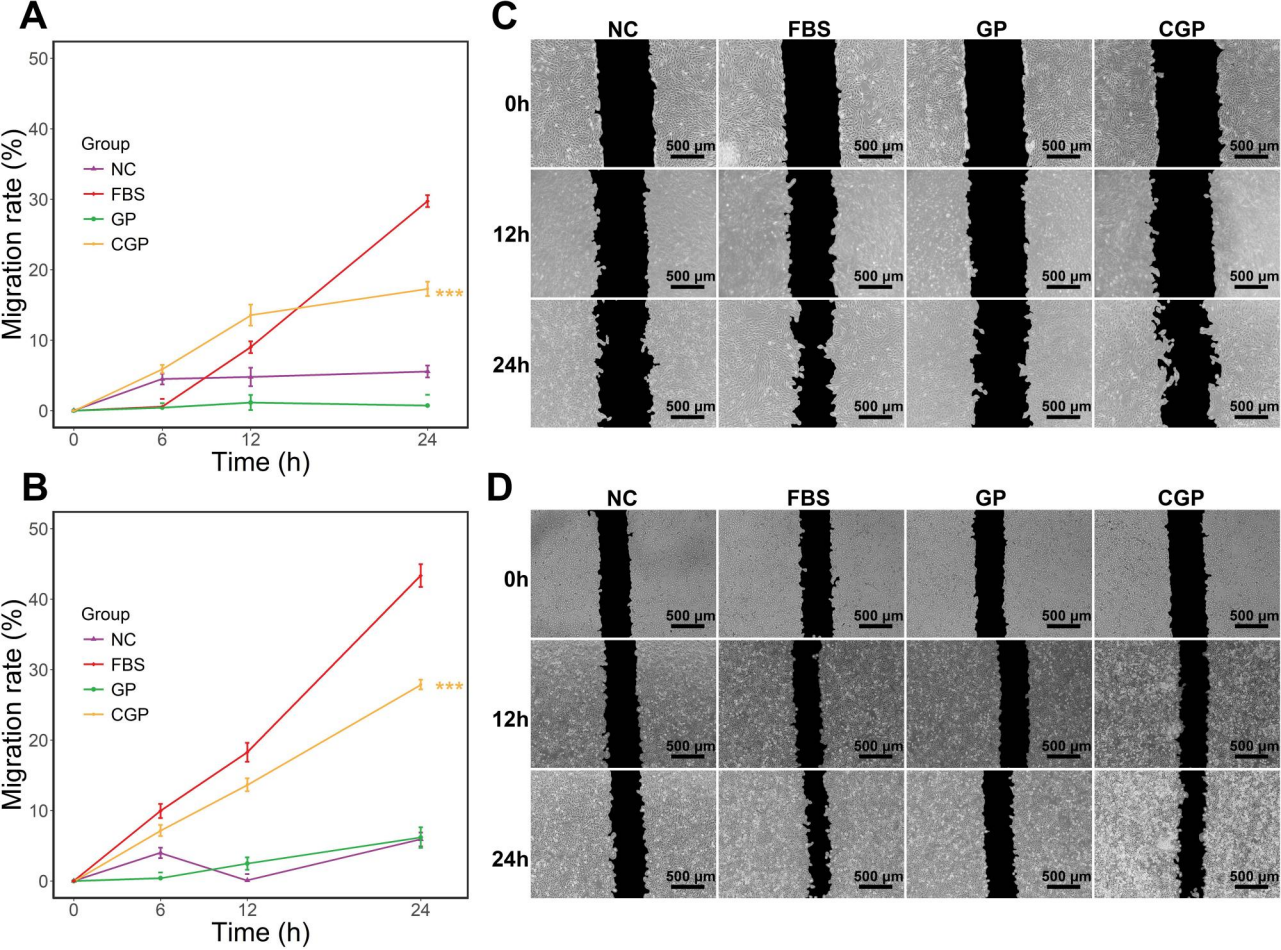
The effect of CGP on C2C12 and HASMCs. The migration rate of (**A**) C2C12 and (**B**) HASMCs were analyzed by two-way ANOVA; representative images of (**C**) C2C12 and (**D**) HASMCs in wound healing assay. (Data was expressed as mean ± SE; * *P* < 0.05, ** *P* < 0.01, *** *P* < 0.001; *n* = 6.)

To validate the safety of CGP, C2C12 cells, HASMCs and HUVECs were cultured in CGP extract prepared with media specific to each cell type. Cell migration, tube formation and proliferation assays were performed to evaluate the impact of CGP on cellular viability. As shown in Figure 3A and B, compared to GP, CGP significantly promoted the migratory activity in both C2C12 and HASMCs. For C2C12, although the migration rate in the CGP group was modest, two-way ANOVA revealed a statistically significant improvement over the GP group (Figure 3A and C). For HASMCs, CGP markedly stimulated migration, with a 4.92-fold increase in migration rate compared to the GP group, and the statistical significance was also validated by two-way ANOVA (Figure 3B and D). Moreover, in the tube formation assay using HUVECs, CGP exhibited no pro-angiogenic effects relative to GP (Supplementary Figure S7A and B). CCK-8 assays for proliferative activity, analyzed via two-way ANOVA, also showed that CGP extracts did not significantly stimulate proliferation in C2C12, HASMCs or HUVECs at the concentrations tested. Observed statistical differences in *t*-tests may reflect Type I errors due to multiple testing (Supplementary Figure S7C–E).

These results revealed that CGP exerted no adverse effects on cellular migration, proliferation or tube formation capability in C2C12, HASMCs or HUVECs and that it even mildly enhanced the migratory activity of C2C12.

### Therapeutic effect of CGP on hindlimb ischemia

To evaluate the therapeutic efficacy of CGP in hindlimb ischemia, we established a murine hindlimb ischemia model by ligating both ends of the femoral artery and cutting the arterial segment in both hindlimbs. On postoperative Day 28, open field testing was performed across all experimental groups.

**Figure 4:**
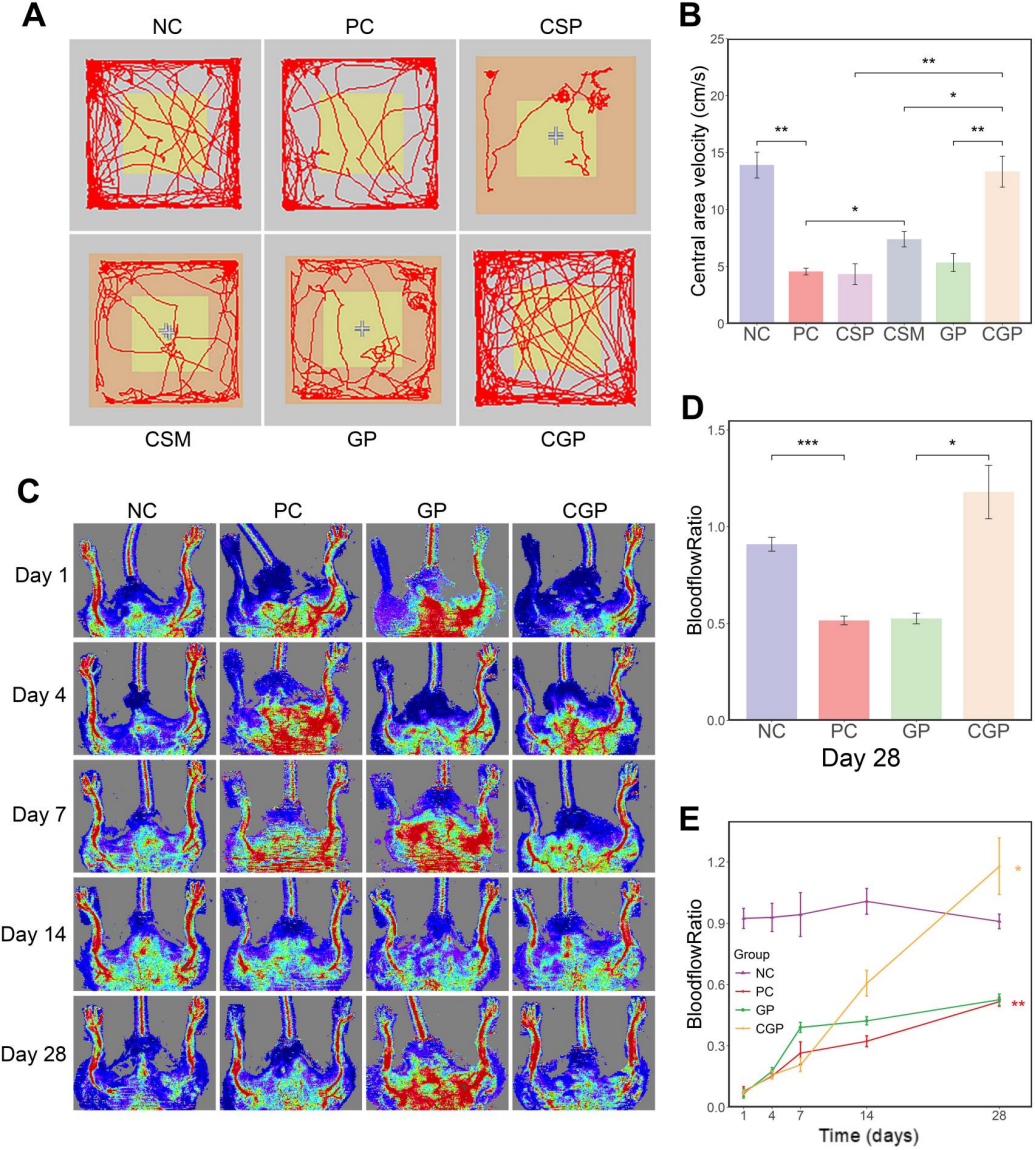
The therapeutic effect of CGP on hindlimb ischemia. (**A**) Travel trajectory maps from open field testing on postoperative Day 28; (**B**) quantitative analysis of central zone velocity during open field test employed *t*-test; (**C**) representative laser Doppler perfusion images of ischemic hindlimbs; (**D**) quantitative analysis of ankle and foot blood perfusion ratio (ischemic/contralateral limb) on Day 28 employed *t*-test; (**E**) line chart depicting ankle perfusion ratio (ischemic/contralateral limb) on postoperative Days 1, 4, 7, 14 and 28 employed two-way ANOVA. (Data was expressed as mean ± SE; * *P* < 0.05, ** *P* < 0.01, *** *P* < 0.001; *n* = 6.)

Analysis of open-field trajectory maps revealed no significant differences in travel distance among groups except for the CSP group, which exhibited reduced locomotion (Figure 4A). Due to the innate thigmotaxis behavior of mice [38], central area velocity was used in this study as a functional indicator of hindlimb capacity. Quantitative analysis demonstrated that the central area velocity in the PC group was significantly lower than that in the NC group. Compared to the PC group, the CSP group showed no improvement, whereas the CSM group exhibited a modest but statistically significant 1.62-fold increase in central area velocity. Notably, the CGP group not only achieved a significant 2.49-fold improvement over the GP group but also outperformed the CSM group in enhancing central area velocity (Figure 4B). These results indicate that, compared with curcumin suspension, curcumin loaded in CGP hydrogel at the same dosage exerted superior therapeutic efficacy, which may be attributed to the sustained release effect of the self-healing hydrogel on the encapsulated drug [20].

Since laser Doppler flowmetry requires self-control, only the left hindlimb ischemia model was established for this assay. This was confirmed on postoperative Day 1 by LDPI, which demonstrated near-absence of blood flow in the ischemic left hindlimb. Subsequent monitoring revealed partial blood flow recovery in the ischemic limbs across all groups over the 28-day experimental period (Figure 4C). No obvious difference was observed among the three experimental groups (except the NC group) until Day 14. Meanwhile, on postoperative Days 1, 4, 7 and 14, these groups showed significantly lower perfusion than the NC group.

On postoperative Day 28, the CGP group exhibited a 2.24-fold improvement in relative blood perfusion ratio compared with the GP group. Notably, the CGP group achieved a relative blood perfusion ratio of 117% by Day 28, showing no significant difference from the NC group. In contrast, the blood flow ratios in the PC and GP groups remained significantly less than those of the NC group (Figure 4D). Longitudinal analysis using two-way ANOVA showed that the ischemic/contralateral ankle-foot blood flow ratio after ligation was consistently lower than that of the NC group throughout the study, and the CGP group demonstrated a significantly improved blood flow ratio relative to the GP group (Figure 4E).

In addition, LDPI revealed no improvement in blood perfusion of the affected limb until postoperative Day 14. Furthermore, as shown in Supplementary Figure S8, no statistically significant differences were observed between the GP and CGP groups in microCT analysis or CD31/α-SMA IF co-staining. These findings, corroborated by the previous tube formation assay using HUVECs, suggested that CGP likely does not promote collateral angiogenesis. Therefore, the observed improvement in perfusion detected by LDPI on Day 28 may instead result from the protective effects of CGP on motor function in ischemic skeletal muscle, which increased the physical activity of model mice, thereby enhancing blood perfusion. Similarly, a 3-month exercise training of supervised high-intensity indoor walking has been observed to significantly improve blood perfusion and oxygen extraction capacity in the calf muscle of the affected limb in patients with PAD [7].

### Downregulation of *Atp6v0d2* by CGP in RNA-seq of gastrocnemius

**Figure 5:**
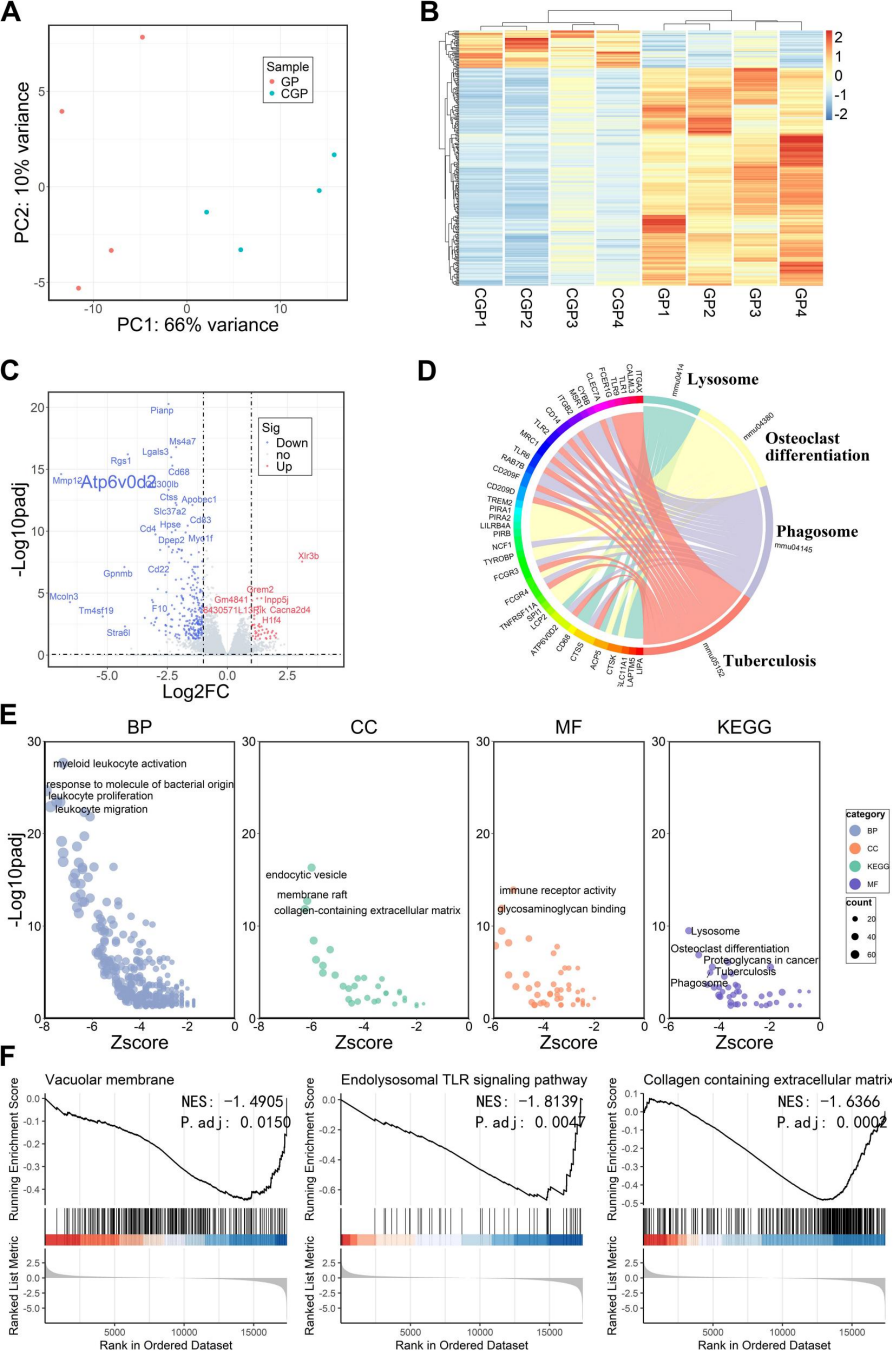
The transcriptomic analysis of ischemic gastrocnemius muscles in hindlimb ischemia model mice treated with CGP versus GP. (**A**) Heatmap between CGP and GP groups; (**B**) PCA plot of CGP and GP groups; (**C**) volcano plot depicting DEGs between GP and CGP groups, with DEGs defined as genes showing |log_2_ (fold change)| >1 and adjusted *P* < 0.05 in RNA-seq; (**D**) chord diagram displaying KEGG pathways enriched with DEGs; (**E**) bubble plots of DEG-enriched pathways from the Gene Ontology database (biological process, cellular component, molecular function) and KEGG database; (**F**) GSEA results for representative pathways. (*n* = 4).

To further investigate the mechanism by which CGP improves hindlimb ischemia in mice, we performed RNA-seq on ischemic gastrocnemius muscle specimens collected from 4 mice in the CGP group and four mice in the GP group on postoperative Day 28. Among the 307 differentially expressed genes (DEGs), 45 were upregulated and 262 downregulated. Principal component analysis (PCA) and heatmap revealed significant transcriptomic differences between the ischemic gastrocnemius muscles of the CGP and GP groups (Figure 5A and B). Volcano plot analysis showed that *Atp6v0d2* was one of the most significant DEGs between these two groups (Figure 5C). DEGs were mapped to the KEGG and GO databases, revealing alterations in multiple signaling pathways in the CGP group, primarily involving lysosomes, osteoclaste differentiation, phagosomes and tuberculosis (Figure 5D and E). Notably, *Atp6v0d2* was involved in three of these pathways. Gene Set Enrichment Analysis (GSEA) further indicated pathways, including the vacuolar membrane, lysosomal lumen and collagen-containing extracellular matrix, were significantly downregulated in the CGP group (Figure 5F).

Atp6v0d2 is a component of Vacuolar-type H+-ATPase (V-ATPase) which regulates autolysosomal pH following autophagosome-lysosome fusion and is consequently essential for autophagic flux [39, 40]. While most studies suggest that enhanced autophagy exerts cytoprotective effects under pathological conditions, our sequencing data revealed a significant transcriptional downregulation of *Atp6v0d2* in CGP-treated ischemic gastrocnemius. Thus, the role of CGP in modulating autophagy and its impact on ischemic skeletal muscle would be elucidated in subsequent experiments.

### The effect of Atp6v0d2 on autophagy in skeletal muscle

**Figure 6:**
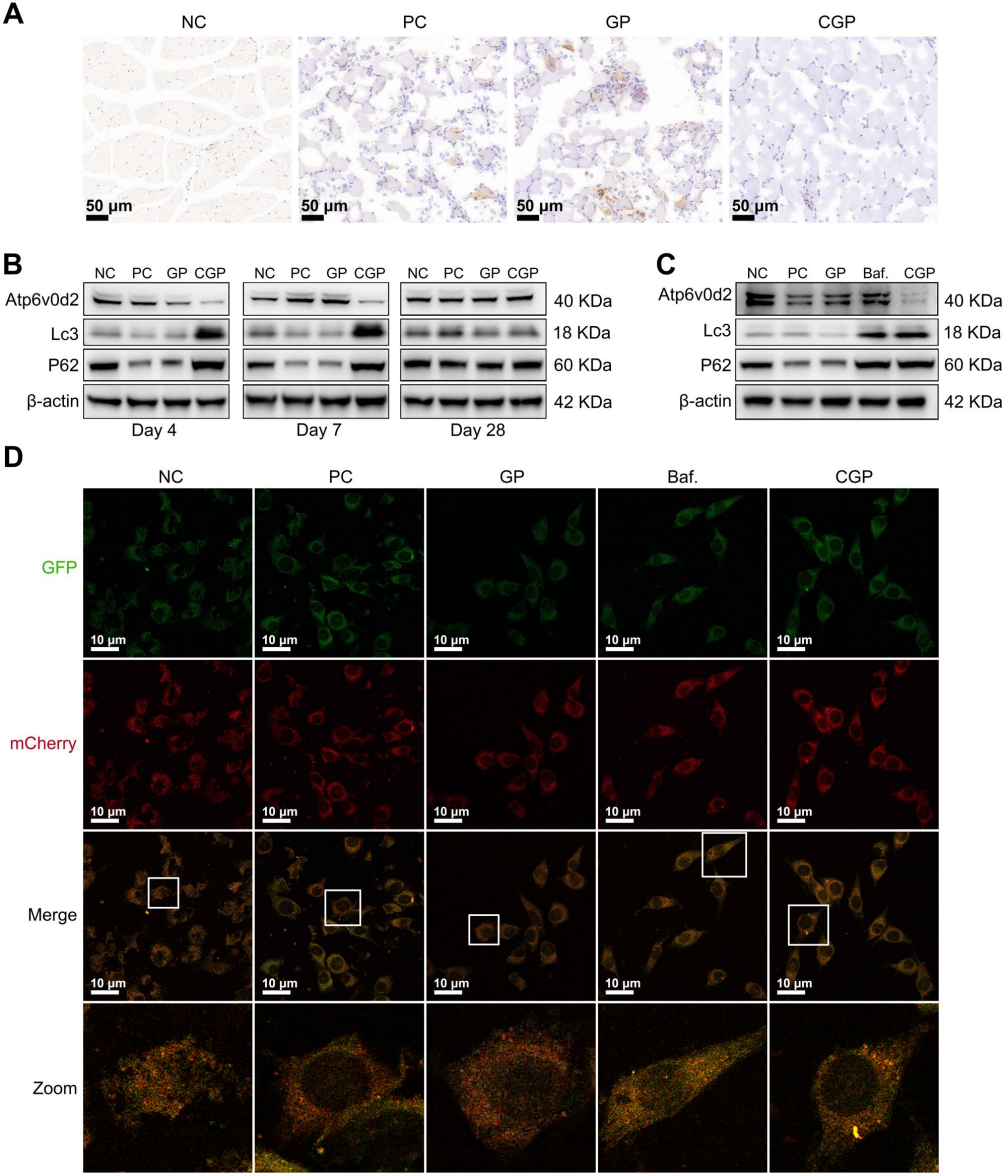
The effect of CGP on autophagy in skeletal muscle. (**A**) Representative images of immunohistochemical staining for Atp6v0d2 on ischemic gastrocnemius sections at postoperative Day 4; (**B**) WB bands of ischemic gastrocnemius muscle at postoperative Day 4, Day 7 and Day 28 (*n* = 6); (**C**) WB bands of C2C12 under OGD conditions (*n* = 6); (**D**) representative confocal images of C2C12 transfected with pCMV-mCherry-GFP-Lc3 plasmids under OGD conditions.

Subsequently, we performed IHC staining for Atp6v0d2 on ischemic gastrocnemius sections on postoperative Day 4. Analysis revealed predominant localization of Atp6v0d2 expression within skeletal muscle cells, with significantly reduced levels observed in the CGP group (Figure 6A). Consistent with these findings, Western blot (WB) analysis of these tissue samples demonstrated persistently lower Atp6v0d2 expression in the CGP group compared with GP groups at both Days 4 and 7 (Figure 6B). Similarly, in C2C12 cells under OGD conditions, significantly downregulated Atp6v0d2 expression in the CGP group was also observed by WB analysis (Figure 6C).

Although Atp6v0d2, as a component of the V0 subunit of V-ATPase, is dispensable for maintaining the acidic environment of autophagolysosomes in macrophages, it plays a critical role in autophagosome-lysosome fusion [41]. To further evaluate the impact of Atp6v0d2 and V-ATPase on autophagy in C2C12, we transfected C2C12 with pCMV-mCherry-GFP-Lc3 plasmids. In this system, GFP quenches in acidic autolysosomes, whereas mCherry remains stable throughout autophagic flux. Confocal imaging revealed that NC, OGD groups (PC) and GP groups exhibited 35–52% yellow puncta (representing co-localized red/green fluorescence). In contrast, both bafilomycin A1-treated (Baf., a V-ATPase inhibitor [42]) and CGP groups showed a marked increase in yellow puncta (79% and 90%, respectively), which suggested the blockade of autophagic flux by Baf. and CGP (Figure 6D and Supplementary Figure S9).

WB analysis of C2C12 cells under OGD conditions revealed the accumulation of Lc3-ii and P62 in both CGP and Baf. groups (Figure 6C). This phenomenon was also observed in ischemic gastrocnemius muscles from the CGP group (Figure 6B). Lc3-ii and P62 are established autophagic markers. Previous studies have indicated that curcumin elevates Lc3-ii levels, leading to the conclusion that its therapeutic effect is mediated through the promotion of autophagy [43]. However, when V-ATPase activity is impaired during autophagy, these proteins accumulate intracellularly due to defective lysosomal hydrolysis within autolysosomes. Therefore, the accumulation of Lc3-ii and P62 induced by CGP under ischemic conditions is attributable to the inhibition of autophagy by CGP.

Furthermore, following lentivirus-mediated modulation of *Atp6v0d2* expression, qPCR analysis revealed increased expression of *P62* in *Atp6v0d2*-knockdown C2C12 cells under OGD conditions. Conversely, in C2C12 cells treated with CGP extract, overexpression of *Atp6v0d2* significantly reduced *P62* expression (Supplementary Figure S10A–D). These results suggest that the inhibitory effect of CGP on autophagy may be mediated, at least in part, by the suppression of Atp6v0d2 expression.

### Protective effect of CGP on the muscles during hindlimb ischemia

Approximately 35% of patients with PAD have comorbid sarcopenia [44]. Sarcopenia is defined as age-related low muscle strength, low muscle quantity/quality and low physical performance [45]. PAD patients with comorbid sarcopenia exhibit significantly lower 5-year overall survival rates and 3-year cardiovascular event-free survival rates compared to the general PAD population [46]. However, aside from thymoquinone and edaravone, few pharmacological agents have been shown to exert direct protective effects on ischemic skeletal muscle [47, 48].

**Figure 7:**
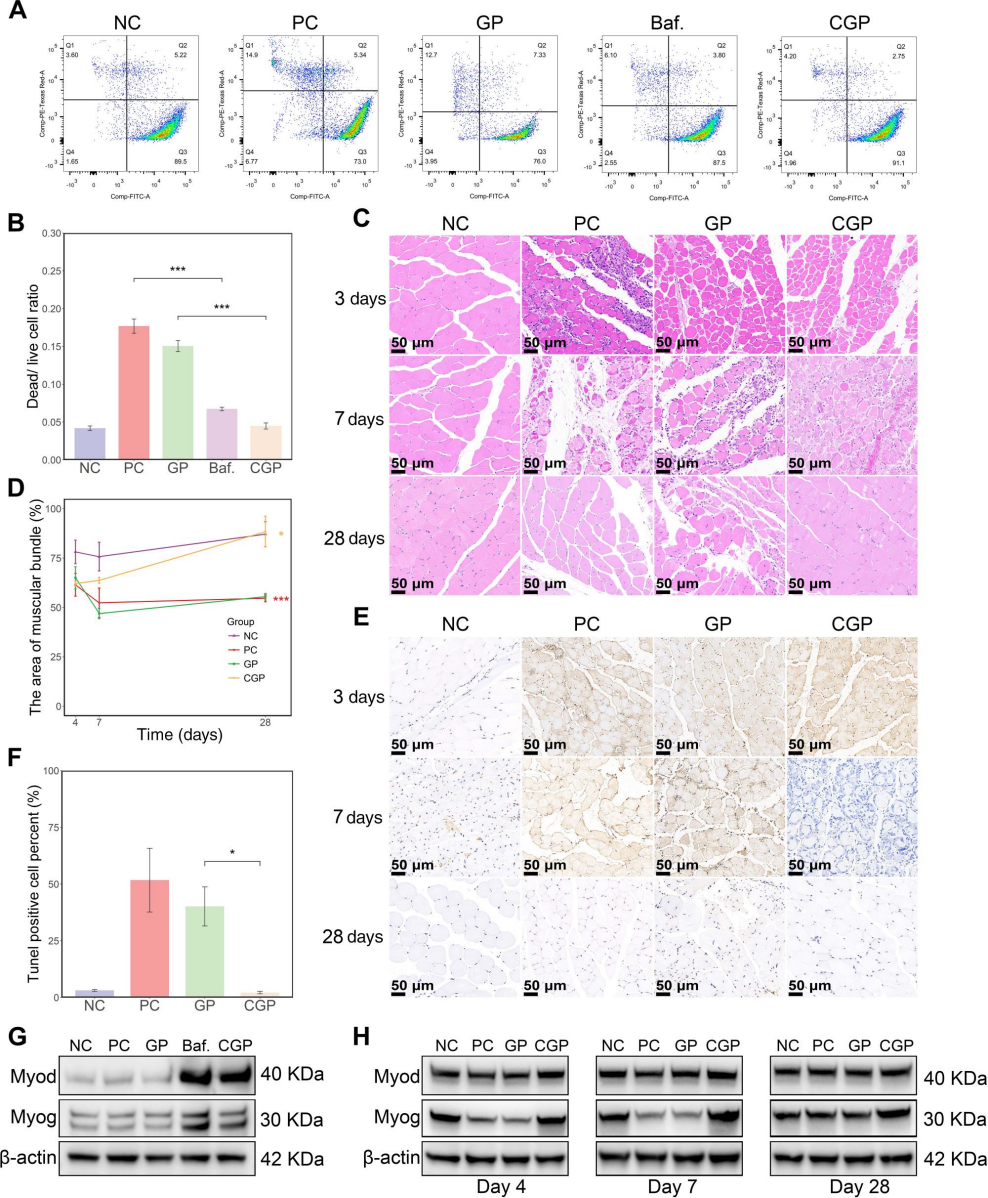
The protective effect of CGP on skeletal muscle. Flow cytometry result of live/dead cell staining of C2C12 cells under OGD condition in different treatment groups (**A**) and quantitative analysis employing *t*-test (**B**). (**C**) Representative H&E-stained images of ischemic gastrocnemius muscles from hindlimb ischemic mice on postoperative Days 4, 7 and 28. (**D**) Line chart showing changes in cross-sectional area of muscle bundles across groups on postoperative Days 4, 7 and 28 employed two-way ANOVA. (**E**) Representative TUNEL-stained images of ischemic gastrocnemius muscles on postoperative Days 4, 7 and 28. (**F**) Quantitative analysis of apoptotic cell percentage at postoperative Day 7 employed *t*-test. (**G**) Representative WB bands of Myod and Myog expression in C2C12 cells under OGD conditions with different treatments. (**H**) Representative WB bands of Myod and Myog expression in ischemic gastrocnemius muscles on postoperative Days 4, 7 and 28. (Data was expressed as mean ± SE; * *P* < 0.05, ** *P* < 0.01, *** *P* < 0.001; *n* = 6).

To validate the protective effects of CGP on skeletal muscle, C2C12 cells were cultured under OGD conditions and treated with Bafilomycin A1 or CGP extract. A significant reduction could be observed compared to the ratio of dead/live cells in the Baf. and CGP groups with that in the PC and GP groups (Figure 7A). This finding indicates that CGP exerts protective effects on C2C12 cells under OGD conditions.

In response to ischemia, not only is apoptosis induced to reduce nutritional demand, but autophagy is also activated to provide raw materials such as sugars, amino acids and fatty acids for essential metabolism and survival, thereby maintaining intracellular homeostasis [49]. Suppressing autophagy through silencing of ULK1, a core autophagy-related protein, significantly exacerbates apoptosis under hypoxic conditions [50]. However, studies in myocardial infarction models have also shown that curbing excessive autophagy can markedly reduce cardiomyocyte apoptosis and decrease infarct size [51]. Furthermore, in ischemia/reperfusion injury, the activation of autophagy during the ischemic phase removes excess metabolic waste and helps ensure cardiomyocyte survival, whereas excessive autophagy during reperfusion depletes cellular components and leads to autophagic cell death [52]. In this study, under OGD conditions, CGP hydrogel exerted a protective effect against C2C12 cell death similar to that of bafilomycin A1 (Figure 7B). These findings suggest that OGD-induced autophagy may exert deleterious effects on cellular survival.

In the animal experiments, Hematoxylin and Eosin (H&E) staining and TUNEL staining were performed on ischemic gastrocnemius muscles harvested on postoperative Days 4, 7 and 28. H.E. staining revealed edema and leukocyte infiltration in the gastrocnemius muscles of the PC group by Day 4. By Day 7, the PC group exhibited reduced leukocyte infiltration but displayed numerous vacuoles and intercellular gaps, indicating partial muscle necrosis or atrophy. In contrast, while edema was observed in both the GP and CGP groups on Day 4, significant leukocyte infiltration only emerged by Day 7. Notably, the CGP group showed milder edema, smaller intercellular gaps and reduced leukocyte infiltration compared with the GP group. By Day 28, persistent intercellular gaps were obvious in the PC and GP groups, whereas the CGP group exhibited minimal intercellular gaps, and the muscle bundle content in the CGP group was comparable to that in the NC group, indicating that CGP administered during the acute phase of hindlimb ischemia protects ischemic myocytes (Figure 7C). Two-way ANOVA of muscle bundle area in the ischemic gastrocnemius demonstrated that the PC groups consistently had smaller muscle bundle areas than the NC group throughout the experiment. Although the CGP group showed reduced muscle bundle area on Days 4 and 7, it recovered to NC group levels by Day 28, with significant improvement relative to the GP group (Figure 7D).

TUNEL staining was performed to assess apoptosis in the ischemic gastrocnemius muscles of mice [53]. On Day 4, significant apoptosis was observed in the PC, GP and CGP groups (Figure 7E). By Day 7, the CGP group exhibited a statistically significant reduction in apoptosis compared with the GP group, with apoptotic cells accounting for only 5.33% of those in the GP group—a level which was comparable to the NC group (Figure 7F). Overall, CGP accelerated the resolution of apoptosis under ischemic conditions, though two-way ANOVA did not reveal a statistically significant difference between the CGP and GP groups over the entire experimental period (Supplementary Figure S11). Additionally, apoptotic cells decreased in all treatment groups by Day 28, likely due to the robust endogenous capillary formation capacity of the C57BL/6 mouse, which protects skeletal myocytes following arterial ligation [54].

Myog has been shown to confer protection against apoptosis in myocytes [55]. WB analysis confirmed that Baf. and CGP markedly upregulated the protein expression of Myod and Myog in C2C12 under OGD conditions *in vitro* (Figure 7G). In murine ischemic gastrocnemius muscles, no obvious difference in Myod protein levels was observed between the CGP and GP groups throughout the 28-day experimental period. But the expression of Myog exhibited marked fluctuations during the study, with markedly higher levels in the CGP group compared to the GP group (Figure 7H).

Furthermore, as shown in Supplementary Figure S10E, the expression of *Myog* and *Myod* under OGD conditions was promoted by knockdown of *Atp6v0d2* in C2C12 cells. Meanwhile, compared with intervention using CGP extract alone, the expression levels of *Myog* and *Myod* were significantly decreased in C2C12 cells overexpressing *Atp6v0d2* (Supplementary Figure S10F). These findings suggest that CGP preserves cellular morphology and inhibits apoptosis in skeletal myocytes under ischemia conditions, likely by suppressing ischemia-induced cellular autophagy.

### Inhibitory effect of CGP on skeletal muscular fibrosis during hindlimb ischemia

In addition to inducing necrosis and apoptosis in skeletal muscle cells, severe physical injury, excessive inflammation or ischemia/reperfusion injury can also trigger skeletal muscle fibrosis [56, 57]. Skeletal muscle fibrosis results from the excessive secretion of extracellular matrix. Moderate extracellular matrix secretion can serve as a scaffold to guide directional regeneration of skeletal myocytes [58]. It is worth noting that the regeneration of skeletal muscle after revascularization is often accompanied by excessive proliferation of fibrous connective tissue, which can impair the functionality of newly formed muscle and disrupt normal myocyte regeneration [59, 60].

**Figure 8:**
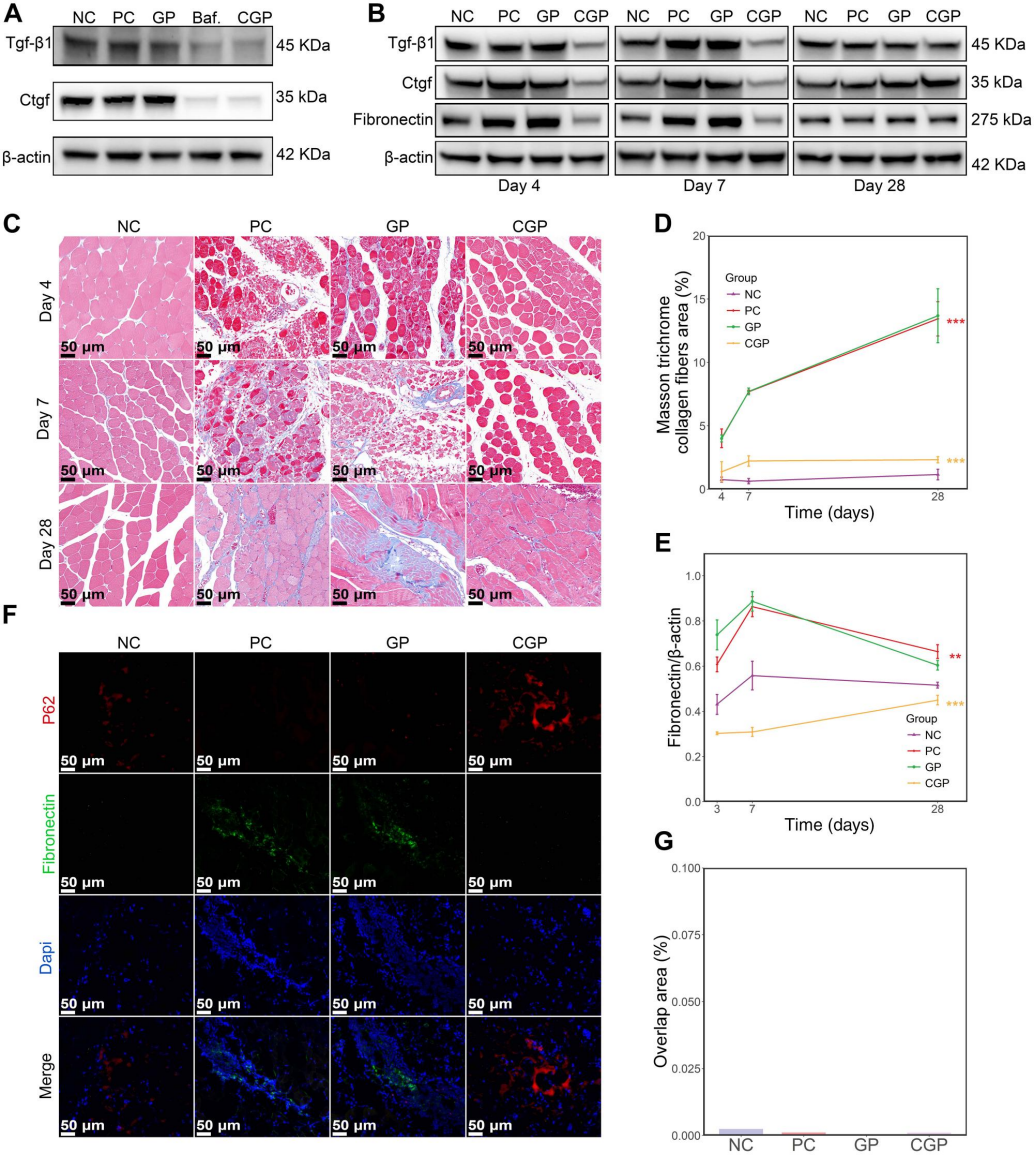
The inhibitory effect of CGP on skeletal muscle fibrosis. (**A**) Representative WB bands of Tgfb1 and Ctgf expression in C2C12 cells under OGD conditions with different treatments. (**B**) Representative WB bands of Tgfb1 and Ctgf expression in ischemic gastrocnemius muscles at postoperative Days 4, 7 and 28; (**C**) Representative Masson’s trichrome-stained images of ischemic gastrocnemius muscles from hindlimb ischemia mouse models on postoperative Days 4, 7 and 28. (**D**) Line chart showing changes in the cross-sectional area of collagen fiber area across groups at postoperative Days 4, 7 and 28 employed two-way ANOVA. (**E**) Line chart showing changes in Fibronectin expression across groups at postoperative Days 4, 7 and 28 employed two-way ANOVA. (**F**) Representative IF-stained images for P62 and Fibronectin in ischemic gastrocnemius muscles from hindlimb ischemia mouse models on postoperative Day 4. (**G**) Semiquantitative analysis of overlap between P62-positive and Fibronectin-positive areas across all groups on postoperative Day 4. (Data was expressed as mean ± SE; * *P* < 0.05, ** *P* < 0.01, *** *P* < 0.001; *n* = 6.)

Under ischemic conditions, CGP not only protects skeletal muscle cells but also inhibits excessive skeletal muscle fibrosis. In C2C12 cells under OGD conditions, although no significant differences were observed in the expression of pro-fibrotic cytokines such as TGFB1 and CTGF between the PC group and NC group, the expression of both cytokines was significantly suppressed in the Baf. group and CGP group (Figure 8A). Similarly, in the ischemic gastrocnemius muscles of mice with hindlimb ischemia, no marked differences in TGFB1 and CTGF expression were detected between the PC and NC groups throughout the experimental period, aside from a minor increase in Ctgf levels. However, on Day 4 and Day 7 of ischemia, the expression of both cytokines was markedly inhibited in the CGP group compared to the GP group (Figure 8B). Significant fluctuations in the expression levels of *Tgfb1* and *Ctgf* were also observed in C2C12 cells following Atp6v0d2 knockdown or overexpression (Supplementary Figure S10C and D).

As skeletal muscle injury persists, affected cells, including myocytes and myoblasts, secrete cytokines such as TGFB1 and CTGF. These cytokines promote collagen secretion by fibroblasts, ultimately leading to scar formation. Therefore, collagen fiber content can, to some extent, indirectly reflect the severity of skeletal muscle injury [61]. In this study, the extent of skeletal muscle fibrosis in the gastrocnemius muscles of mouse ischemic limbs was evaluated using Masson’s trichrome staining. On postoperative Days 4, 7 and 28, blue-stained collagen fiber deposition was observed in the ischemic gastrocnemius muscles of all groups except the NC group. The PC group and GP group consistently exhibited significantly higher collagen deposition than the NC and CGP groups (Figure 8C). Two-way ANOVA revealed statistically significant differences in collagen fiber content between the PC and NC groups throughout the observation period. Moreover, the CGP group showed significantly lower collagen fiber content than the GP group over the entire observation period, and this difference progressively widened over time (Figure 8D).

In addition to collagen protein, fibronectin is another key extracellular matrix component involved in skeletal muscular fibrosis [62]. In the ischemic gastrocnemius muscles, a pattern consistent with the Masson staining results was observed. Throughout the experimental period, Fibronectin levels in the PC group were significantly increased compared with those in the NC group. In contrast, fibronectin expression in the CGP group was markedly suppressed relative to the GP group (Figure 8B). Two-way ANOVA also revealed statistically significant differences between the NC and PC groups, as well as between the GP and CGP groups (Figure 8E). These results demonstrate that CGP can suppress skeletal muscle fibrosis induced by limb ischemia.

Finally, IF staining for P62 and Fibronectin in ischemic gastrocnemius muscles showed significantly larger P62-positive areas in the CGP group compared with the other three groups (Figure 8F and Supplementary Figure S12A). This finding suggests that CGP leads to P62 accumulation by inhibiting autophagic flux. Concurrently, the PC and GP groups exhibited significantly larger fibronectin-positive areas than the other groups, which is consistent with previous findings (Figure 8F and Supplementary Figure S12B). Notably, minimal co-localization was observed between P62-positive and Fibronectin-positive areas across all groups, further suggesting that ischemia-induced autophagy is associated with skeletal muscle fibrosis (Figure 8F and G). Together, these results indicate that CGP may inhibit skeletal muscle fibrosis by suppressing ischemia-induced autophagy.

## Conclusion

In this study, a bioactive GP hydrogel incorporating curcumin was developed, and its safety profile was validated through *in vitro* experiments. *In vivo* investigations demonstrated that local injection of CGP into ischemic hindlimbs provided significantly superior protection of motor function in mice compared with curcumin suspension. This unique therapeutic efficacy is attributed to the sustained and effective release of curcumin from the hydrogel. Further, the CGP can protect ischemic skeletal muscle cells and inhibit skeletal muscle fibrosis, which is associated with the inhibitory role of the released curcumin on ischemia-induced autophagy.

## Supplementary Material

rbag103_Supplementary_Data

## Data Availability

Data will be made available on request.
