# Supplementary material for: An injectable self-healing curcumin-sustained-release hydrogel for lower limb ischemia via autophagy inhibition by Atp6v0d2 downregulation
Source: Regen Biomater. 2026 Jul 2;13:rbag103. doi: 10.1093/rb/rbag103 (PMC13447015; doi:10.1093/rb/rbag103)
Supplement: Supplementary file 1 [file rbag103_supplementary_data.docx]

# An Injectable Self-Healing Curcumin-Sustained-Release Hydrogel for Lower Limb Ischemia via Autophagy Inhibition by Atp6v0d2 Downregulation

Hanfei Tang ^1, †^, Fang Wang ^2, 3, †^, Tonglei Han ^1, †^, Xuanyong Liu ^2^, Minhui Li ^1^, Jiaqi Zhu ^1, *^, Xiao Tang ^1, *^, Guowei Liu ^4, *^, Jiajun Qiu ^2, *^, Daqiao Guo ^1, *^

**Fig. S1.** Representative photographs depicting the sol-gel transformation of the GP hydrogel.

**Fig. S2.** Step-strain measurements (between 1 and 500% strain) to evaluate the self-healing properties of the GP hydrogel. G': storage modulus. G'': loss modulus .

**Fig. S3.** The the injectable properties of the GP hydrogel. (a) Representative photographs depicting the injectable properties of the GP hydrogel. (b) The displacement-pressure curve of syringe containing GP hydrogel.

**Fig. S4.** The storage modulus (G') and loss modulus (G'') of the GP hydrogel in the frequency range of 0.1-100 Hz (a) and at strains ranging from 1 to 1000% (b).

**Fig. S5.** The standard curve of curcumin solution at the absorbance of 430 nm. The equation of the standard curve is y=30.18x-1.85, (R^2^=0.999).

**Fig. S6.** UV-Vis spectra of DPPH and DPPH treated with 100 μL of ddH_2_O, as well as curcumin suspensions at concentrations of 35 μg mL^-1^、70 μg mL^-1^、175 μg mL^-1^、350 μg mL^-1^.

**Fig. S7.** Representative images of tube formation (A) and quantitative analysis of master junctions (B) in HUVECs treated with ECM, GP extract, or CGP extract. CCK-8 assay results of C2C12 (C), HASMCs (D), and HUVECs (E) cultured in: standard medium, GP extract, or CGP extracts at volume ratios of 10:1000, 25:1000, and 50:1000 for 1, 3, and 5 days.

**Fig. S8.** Collateral angiogenesis of CGP on hindlimb ischemia. (a) CT angiography image of ischemic limb Scanning with micro CT on postoperative day 28. (b) Quantitative analysis of the volume of blood vesssels obtained from micro CT employed t-test. (c) Representative immunofluorescence stained images for CD31 and α-SMA in ischemic gastrocnemius muscles from hindlimb ischemia mouse models at postoperative days 28. (d) Quantitative analysis of average vessel numbers per mm^3^ obtained from CD31/α-SMA immunofluorescence co-stain images employed t-test. (Data was expressed as Mean ± SE; * p< 0.05; n = 6)

**Fig. S9.** Semiquantitative analysis of C2C12 transfected with mCherry-GFP-LC3 plasmids under OGD condition. (n = 6)

**Fig. S10.** Quantitative PCR analysis for mRNA expression in C2C12 cells following lentivirus-mediated modulation of *Atp6v0d2* expression. (a and b) mRNA expression of *Atp6v0d2* in C2C12 cells following *Atp6v0d2* knockdown by LV-shAtp6v0d2 vector (a) and *Atp6v0d2* overexpression by LV- Atp6v0d2 vector (b) . (c, e and g) mRNA expression of *Lc3-ii* & *P62* (c), *Myod* &*Myog* (e), and *Tgfb1* & *Ctgf* (g)in C2C12 cells following *Atp6v0d2* knockdown under ODG condition. (d, f and h) mRNA expression of *Lc3-ii* & *P62* (d), *Myod* & *Myog* (f), and *Tgfb1* & *Ctgf* (h)in C2C12 cells following *Atp6v0d2* overexpression under ODG condition and treated with hydrogel extracts.((Data was expressed as Mean ± SE; Student t-test was employed; * p< 0.05, ** p< 0.01, *** p< 0.001; n = 6)

**Fig. S11.** Quantitative analysis of Tunel staining in ischemic gastrocnemius muscles of hindlimb ischemia mice on postoperative days 4, 7, and 28.

**Fig. S12.** Semi-quantitative analysis of immunofluorescence stained images for P62 and Fibronectin in ischemic gastrocnemius muscles from hindlimb ischemia mouse models at postoperative days 4. (a) the proportion of P62- positive areas in all group. (b) the proportion of Fibronectin- positive area in all group.

| **primer** | **sequence** |
| --- | --- |
| Atp6v0d2-F | GGGCCAGTGTTCAGTTGCTA |
| Atp6v0d2-R | AGTCCGTGGTCTGGAGATGA |
| Lc3-F | GAGGGGACCCTAACCCCATA |
| Lc3-R | CACTTCGGAGATGGGAGTGG |
| P62-F | GCTCTTCGGAAGTCAGCAAACC |
| P62-R | GCAGTTTCCCGACTCCATCTGT |
| Myod-F | AAGACGACTCTCACGGCTTG |
| Myod-R | GCAGGTCTGGTGAGTCGAAA |
| Myog-F | TACAGACGCCCACAATCTGC |
| Myog-R | TCAGTTGGGCATGGTTTCGT |
| Tgfb1-F | ACTGGAGTTGTACGGCAGTG |
| Tgfb1-R | GGGGCTGATCCCGTTGATTT |
| Ctgf-F | AGAACTGTGTACGGAGCGTG |
| Ctgf-R | GTGCACCATCTTTGGCAGTG |

**Supplementary Table 1**. The primer used in RT- qPCR.
